# Applying the Bispectral Analysis on Widespread Diffuse Cross-Frequency Interactive Effects

**DOI:** 10.1155/2013/412802

**Published:** 2013-12-10

**Authors:** Chia-Ju Liu, Cheng-Hsieh Yu, Chin-Fei Huang, Ray-Ying Huang, Chung-Jung Wang, Yi-Shan Liu, Tsung-Ching Chen, Ming-Chung Ho

**Affiliations:** ^1^Graduate Institute of Science Education, National Kaohsiung Normal University, Kaohsiung 824, Taiwan; ^2^Department of Physics, National Kaohsiung Normal University, Kaohsiung 824, Taiwan

## Abstract

The aim of this paper is applying the bispectral analysis on widespread diffuse cross-frequency interactive effects. The event-related potentials (ERPs) research method was used in this study and it could collect the widespread diffuse cross-frequency from mild cognitive impairment (MCI) patients' brain wave. In this study, the brain wave data were collected from 12 MCI subjects, 12 healthy elderly, and 12 healthy young. The findings showed that the decreased interhemispheric coherence of 8.8 Hz for MCI compared with healthy elderly in the central-parietal cortex to respective surrounding sites and each MCI subject showed significantly widespread diffuse pattern of cross-frequency interactions in comparison with the healthy controls in the left central-parietal and right frontal. This study provides some explanation and suggestions for these findings.

## 1. Introduction

Looking for a reliable and sensitive method to identify more accurately mild Alzheimer's disease (AD) patients is one of the challenges of the current research. Now, the research method about event-related potentials (ERPs) provides a noninvasive electrophysiological measure as the earliest markers of mild AD. The past studies demonstrated that the P300 amplitude was relatively smaller and P300 latency was longer for AD compared to healthy controls [1–4]. Hence, the P300 which is a component of ERPs data was supposed to be used as an indicator to detect mild AD.

The previous study mentioned that the auditory oddball paradigm is related to “focused attention,” “target recognition,” “motor response,” “signal detection,” “working memory,” “executive functions,” and “decision making” [5]. The auditory oddball task is an easy task for mild AD patients to respond [4]. Despite the group differences in the auditory oddball task condition, P300 amplitude and latency are not yet sensitive enough to discriminate between mild AD and normal aging [4]. The event-related oscillatory activity in various frequency bands may reflect different aspects of physiological information processing. Also, the oscillatory changes are basic phenomena during cognitive performance [6].

The construct of dementia has been proposed to designate an early, but abnormal, state of cognitive impairment [7]. Mild AD is supposed to represent a substantial proportion of patients with Alzheimer disease [8]. But it lacks the sensitive indicator to diagnose the mild AD. The hemispheric cooperation model [9, 10] is the study conception underlying the compensation view of bilateral activation in healthy elderly. The previous studies also provided different kinds of evidence [11–13], including behavioral data [14], recovery from brain damage [15], and comparison between high and low performance in healthy elderly [11], to compare with the mild AD. Contrasting with this age-related cognitive decline are reliable increases in frontal lobe activation when performing cognitive tasks in old compared to young adults [16], and these may reflect an age-related difficulty in activating the appropriate brain networks to a level that would be sufficient to successfully perform the auditory oddball task.

The purpose of this paper is generaizing the results of past researches and applying the bispectral analysis on widespread diffuse cross-frequency interactive effects on ERPs data from healthy and mild AD patients.

## 2. Materials and Methods 

The behavior data of the subjects are shown in Table 1. MCI participants had lower MMSE (16.9 ± 6.7), longer reaction time (511.5 ± 203.2 ms), and lower correct rate (95.4 ± 10.1) compared to healthy elderly and healthy young. As expected, there was no significant difference on age between MCI and healthy elderly (*P* < 0.05).

MCI was recruited according to NINCDS-ADRDA criteria. All MCI patients underwent general neurological assessments. Patients were also assessed with a number of standardized diagnostic tests, including the Mini Mental State Examination (MMSE) score. None of the participants showed hearing loss, neurological or psychological problems, and all were naive to electrophysiological studies. The study protocol was approved by the medical ethics committee of the *National Kaohsiung Normal University*. All participants were required to give informed consent before taking part in the experiment.

An auditory oddball paradigm was used in the experiments. Two types of stimuli were used: the standards and the deviants. The probability of the deviant stimuli was 0.20 and that of standard stimuli 0.80. As stimulation we used a 2 kHz for standard signals. The 1 kHz of the deviant stimuli was 20% and only deviant trials to which both patients and controls used a button press response. The rise time of the stimulation signal was 50 ms and the duration of the stimulation was 2000 ms. In all the paradigms, the deviant stimuli were embedded randomly within a series of standard stimuli. During the elicitation period of event-related oscillations, all subjects had achieved minimum 95% accuracy of target stimuli on EEG data, with being generally worse in mild AD subjects than in controls. The EEG recordings were performed from 32 electrodes positioned according to the International 10–20 system (i.e., FP1, FP2, F7, F3, Fz, F4, F8, FT7, FC3, FCz, FC4, FT8, T3, C3, Cz, C4, T4, TP7, CP3, CPz, CP4, TP8, T5, P3, Pz, P4, T6, O1, Oz, and O2). The signal was analog-filtered (0.1–200 Hz), A/D-converted with a sampling rate of 1000 Hz and 14 bit precision, and digitally filtered in the range 1–100 Hz.

The previous studies have used bispectral analysis to obtain useful information of anesthetics and neuroactive drugs by observing EEG changes in cerebral functions [17, 18]. The analysis of nonlinear cross-frequency phase synchronization focuses on the functional interplay between different electrodes. Bispectral analysis is effective in frequency domain analysis and for detecting nonlinear interactions of different frequency components [19, 20]. Interconnectivity in frequencies is most likely the neural activity in different cell assemblies, and neuronal excitability may be reflected in the phase difference [21]. The bispectrum is defined by Nikias and Petropulu [17]. The index BIC is the strength of phase coupling of the two signals at a specific frequency (*f*_1_ versus *f*_2_). The BIC values fall into the range of $\left[\begin{array}{cc} 0 & 1 \end{array}\right]$. The criterion *F*_0.95_(BIC (*f*_1_, *f*_2_)) of the bicoherence can be approximated [22]. The EEG data were fragmented offline in 2048 ms epochs and performed at 1024 bins (sampling frequency = 1000 samples/s) in the average. The criterion for significance at the 95% confidence level is 0.063 for the phase bicoherence in this study. The phase coupling between oscillations with frequencies *f*_1_ and *f*_2_ is considered statistically significant when BIC exceeds the threshold for the frequency pair.

## 3. Results and Discussion

**Figure 1:**
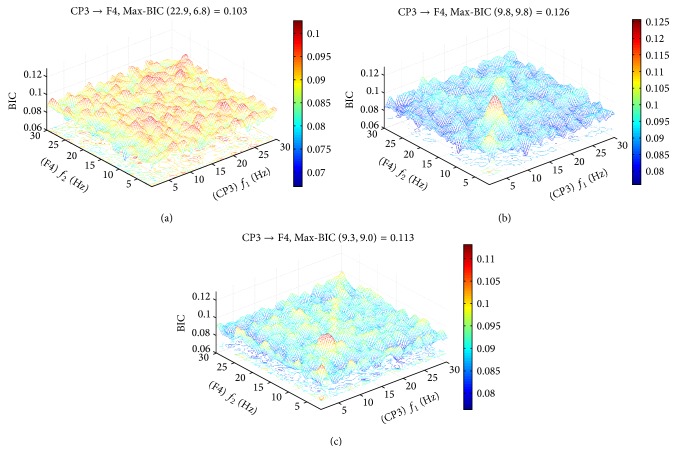
Phase coupling between different frequencies components. Grand average bicoherence repeated 150 trials for each subject. Cross-frequency phase coupling was computed between the signals in electrode central-parietal (*x*-axis) and right frontal (*y*-axis) during the auditory oddball stimuli. The peak for healthy controls indicates a phase self-frequency coupling from 8 to 11 Hz activity in central-parietal and right frontal. (a) Pattern of mild cognitive impairment (Max-BIC (*f*_1_ = 22.9, *f*_2_ = 6.8) = 0.103); (b) pattern of healthy elderly matched for age with MCI (Max-BIC (*f*_1_ = 9.5, *f*_2_ = 9.5) = 0.113); (c) pattern of healthy young (Max-BIC (*f*_1_ = 9.8, *f*_2_ = 9.8) = 0.126). The red circle indicates the maximal phase coupling value in the frequency range 1–30 Hz. This peak (bicoherence at 8–11 Hz) was significant with *P* < 0.05.

Coherence is a linear measure of the correlations between two signals. The coherence provides frequency domain information about the functional connections between different cortical areas.  Figure 1(a) depicts the grand average bicoherence between left central-parietal (CP3) and right frontal (F4) electrodes repeated 150 trials for healthy young. The dominant frequency in the maximal self-coupling spectrum was determined within frequency range 8–11 Hz in Figure 1(b), and only about quarter of the subjects fall into the delta band, while MCI subjects were not found. Although very small diffuse cross-frequency coupling was found for healthy elderly, significant self-frequency coupling exists in Figure 1(c).

The main effect of stimulus was found for MCI, signifying disappeared 8–11 Hz self-coupling, and replaced by a widespread and diffuse cross-frequency coupling in the long-range bicoherence changes.

The results demonstrated widespread diffuse cross-frequency coupling for MCI compared to healthy elderly and healthy young during the performance of an auditory attention task. According to our findings the healthy controls showed that self-coupling is prominent within the range 8–11 Hz. Besides, bicoherence in MCI was found to be less than healthy controls, providing further support in terms of the widespread diffuse coupling distribution in MCI. It is to be emphasized that these changes were detected in the early stage of mild AD. The study of individual differences of the maximum bicoherence value decreased in MCI, suggesting that coupling with the dominant low alpha frequency (8–11 Hz) is a very strong indicator of nonlinearity.

## 4. Conclusion

In this study, the results demonstrated the widespread diffuse cross-frequency coupling in MCI during performance on the auditory oddball task. The increase of coupling may reflect compensatory activity as well as dedifferentiation processes in the long-distance coherence or synchronization. The results implied that a dementia-related functional breakdown in the long-distance coherence or synchronization may be predicted on mild AD diagnosis. The results of this study suggest the increase of cross-frequency coupling in right frontal cortex and central-parietal regions might be an indicator to diagnose mild AD and the increasing processing may reflect the poorer neural function.

## Figures and Tables

**Table 1:** Characteristics of study sample (MCI, healthy elderly and healthy young).

Variable	MCI	Healthy elderly (HE)	Healthy young (HY)	*P* (HE versus MCI)	*P* (HE versus HY)
Age	80.9 ± 8.4	73.9 ± 9.9	20.4 ± 3.1	0.09	0.00∗∗
MMSE total score	16.9 ± 6.7	30 ± 0	30 ± 0		
RT (ms)	511.5 ± 203.2	426.8 ± 77.3	361.8 ± 55.3	0.22	0.04∗
Correct rate (%)	95.4 ± 10.1	98.2 ± 2.8	99.2 ± 2.0	0.27	0.41
Amplitude (*μ*V)	4.5 ± 1.4	10.5 ± 4.4	12.3 ± 4.1	0.00∗∗	0.48
Latency (ms)	405.6 ± 33.8	385.3 ± 36.6	326.1 ± 31.1	0.26	0.01∗

Note: RT means reaction time; ∗*P* < 0.05; ∗∗*P* < 0.01.

## References

[1] Patterson J. V., Michalewski H. J., Starr A. (1988). Latency variability of the components of auditory event-related potentials to infrequent stimuli in aging, Alzheimer-type dementia, and depression. *Electroencephalography and Clinical Neurophysiology*.

[2] Polich J., Ehlers C. L., Otis S. (1986). P300 latency reflects the degree of cognitive decline in dementing illness. *Electroencephalography and Clinical Neurophysiology*.

[3] Polich J., Kok A. (1995). Cognitive and biological determinants of P300: an integrative review. *Biological Psychology*.

[4] Polich J., Corey-Bloom J. (2005). Alzheimer's disease and P300: review and evaluation of task and modality. *Current Alzheimer Research*.

[5] Basar-Eroglu C., Basar E., Demiralp T., Schurmann M. (1992). P300-response: possible psychophysiological correlates in delta and theta frequency channels. A review. *International Journal of Psychophysiology*.

[6] Başar-Eroglu C., Demiralp T. (2001). Event-related theta oscillations: an integrative and comparative approach in the human and animal brain. *International Journal of Psychophysiology*.

[7] Petersen R. C. (2004). Mild cognitive impairment as a diagnostic entity. *Journal of Internal Medicine*.

[8] Morris J. C., Storandt M., Miller J. P. (2001). Mild cognitive impairment represents early-stage Alzheimer disease. *Archives of Neurology*.

[9] Brown W. S., Jeeves M. A. (1993). Bilateral visual field processing and evoked potential interhemispheric transmission time. *Neuropsychologia*.

[10] Weissman D. H., Banich M. T. (2000). The cerebral hemispheres cooperate to perform complex but not simple tasks. *Neuropsychology*.

[11] Cabeza R., Anderson N. D., Locantore J. K., McIntosh A. R. (2002). Aging gracefully: compensatory brain activity in high-performing older adults. *NeuroImage*.

[12] Cabeza R., Grady C. L., Nyberg L. (1997). Age-related differences in neural activity during memory encoding and retrieval: a positron emission tomography study. *Journal of Neuroscience*.

[13] Phillips L. H., Andrés P. (2010). The cognitive neuroscience of aging: new findings on compensation and connectivity. *Cortex*.

[14] Reuter-Lorenz P. A., Stanczak L., Miller A. C. (1999). Neural recruitment and cognitive aging: two hemispheres are better than one, especially as you age. *Psychological Science*.

[15] Feydy A., Carlier R., Roby-Brami A. (2002). Longitudinal study of motor recovery after stroke: recruitment and focusing of brain activation. *Stroke*.

[16] Cabeza R. (2002). Hemispheric asymmetry reduction in older adults: the HAROLD model. *Psychology and Aging*.

[17] Nikias C. L., Petropulu A. P. (1993). *Higher-Order Spectral Analysis: A Nonlinear Signal Processing Framework*.

[18] Swami A., Mendel C. M., Nikias C. L.

[19] Freye E., Levy J. V. (2005). Cerebral monitoring in the operating room and the intensive care unit: an introductory for the clinician and a guide for the novice wanting to open a window to the brain. *Journal of Clinical Monitoring and Computing*.

[20] Schack B., Vath N., Petsche H., Geissler H.-G., Möller E. (2002). Phase-coupling of theta-gamma EEG rhythms during short-term memory processing. *International Journal of Psychophysiology*.

[21] Sauseng P., Klimesch W., Gruber W. R., Birbaumer N. (2008). Cross-frequency phase synchronization: a brain mechanism of memory matching and attention. *NeuroImage*.

[22] Shils J. L., Litt M., Skolnick B. E., Stecker M. M. (1996). Bispectral analysis of visual interactions in humans. *Electroencephalography and Clinical Neurophysiology*.

